# Recurrence of cystic echinococcosis in an endemic area: a retrospective study

**DOI:** 10.1186/s12879-017-2556-9

**Published:** 2017-06-27

**Authors:** Virginia Velasco-Tirado, Ángela Romero-Alegría, Moncef Belhassen-García, Montserrat Alonso-Sardón, Carmen Esteban-Velasco, Amparo López-Bernús, Adela Carpio-Perez, Marcelo Fernando Jimenez López, Juan Luis Muñoz Bellido, Antonio Muro, Miguel Cordero-Sanchez, Javier Pardo-Lledias, Luis Muñoz-Bellvis

**Affiliations:** 1 Servicio de Dermatologia Complejo Asistencial Universitario de Salamanca (CAUSA), Instituto de investigación Biomédica de Salamanca (IBSAL), Centro de Investigación de Enfermedades Tropicales de la Universidad de Salamanca (CIETUS), Universidad de Salamanca, Paseo San Vicente, Salamanca, Spain; 2 Servicio de Medicina Interna, CAUSA, IBSAL, CIETUS, Universidad de Salamanca, Paseo San Vicente, 58-182, 37007 Salamanca, Spain; 3 Servicio de Medicina Interna, Sección de Enfermedades Infecciosas, CAUSA, IBSAL, CIETUS, Universidad de Salamanca, Paseo San Vicente, 58-182, 37007 Salamanca, Spain; 4 Área de Medicina Preventiva y Salud Pública, IBSAL, Universidad de Salamanca, Salamanca, Spain; 5 Servicio de Cirugia, CAUSA, IBSAL, Universidad de Salamanca, Salamanca, Spain; 6 Servicio de Medicina Interna, CAUSA, IBSAL, CIETUS, Salamanca, Spain; 7 Servicio de Cirugía Torácica, CAUSA, IBSAL, Universidad de Salamanca, Salamanca, Spain; 8 Servicio de Microbiología CAUSA, IBSAL, Universidad de Salamanca, Salamanca, Spain; 9 Laboratorio de Inmunología Parasitaria y Molecular, CIETUS, IBSAL, Facultad de Farmacia, Universidad de Salamanca, Salamanca, Spain; 10 Servicio de Medicina Interna, Seccion de Enfermedades Infecciosas, CAUSA, CIETUS, IBSAL, Universidad de Salamanca, Salamanca, Spain; 11 Servicio de Medicina Interna, Hospital General de Palencia “Río Carrión”, C/ Donantes de Sangre, Palencia, Spain

**Keywords:** Cystic echinococcosis, *Echinococcus granulosus*, Hydatidosis, Recurrence, Treatment

## Abstract

**Background:**

Cystic echinococcosis (CE) is a chronic, complex and neglected zoonotic disease. CE occurs worldwide. In humans, it may result in a wide spectrum of clinical manifestations, ranging from asymptomatic infection to fatal disease. Clinical management procedures have evolved over decades without adequate evaluation. Despite advances in surgical techniques and the use of chemotherapy, recurrence remains one of the major problems in the management of hydatid disease. The aim of this study was to determine the frequency of CE recurrence and the risk factors involved in recurrence.

**Methods:**

A descriptive longitudinal-retrospective study was designed. We reviewed all patients diagnosed with CE according to ICD-9 (code 122–0 to 122–9) criteria admitted at Complejo Asistencial Universitario de Salamanca, Spain, between January 1998 and December 2015.

**Results:**

Among the 217 patients studied, 25 (11.5%) had a hydatid recurrence after curative intention treatment. Median duration of recurrence’s diagnosis was 12.35 years (SD: ±9.31). The likelihood of recurrence was higher [OR = 2.7; 95% CI, 1.1–7.1; *p* < 0.05] when the cyst was located in organs other than liver and lung, 22.6% (7/31) vs 14.2% (31/217) in the cohort. We detected a chance of recurrence [OR = 2.3; 95% CI, 1.4–6.5; *p* > 0.05] that was two times higher in those patients treated with a combination of antihelminthic treatments and surgical intervention (20/141, 14.2%) than in patients treated with surgical intervention alone (5/76, 6.6%).

**Conclusions:**

Despite advances in diagnosis and therapeutic techniques in hydatid disease, recurrence remains one of the major problems in the management of hydatid disease. The current management and treatment of recurrences is still largely based on expert opinion and moderate-to-poor quality of evidence. Consequently, large prospective and multicenter studies will be needed to provide definitive recommendations for its clinical management.

## Background

Cystic echinococcosis (CE) is a chronic, complex and neglected zoonotic disease caused by the larval stage (metacestode) of *Echinococcus granulosus*. CE occurs worldwide but is endemic to central Asia, northern and eastern Africa, Australia, South America and the Mediterranean Basin [1–3]. CE has peculiar features that imply difficulties in the evaluation of its magnitude [2]. In humans, it may result in a wide spectrum of clinical manifestations, ranging from asymptomatic infection to fatal disease [4]. Its clinical management is complex, has evolved over decades without adequate evaluation of efficacy, effectiveness, rate of adverse reactions, relapse rate, and cost. The current management and treatment of CE is still largely based on expert opinion and moderate-to-poor quality of evidence [5, 6]. Despite advances in surgical techniques and the use of chemotherapy, recurrence remains one of the major problems in the management of hydatid disease [7]. Overall, CE recurrence rates appear to be highly variable (0%–22%) and are seen in intervals ranging from 3 months to 20 years from the first operation [8]. The recurrence of disease may present with major complications including pyogenic infection, intrabiliary rupture, or anaphylaxis. However, it is usually initially asymptomatic, and therefore regular long-term follow-up should be routine after primary treatment [9, 10]. Recurrent disease is the main criteria of failure of treatment [10]. Finally, the absence of protocols and clinical guidelines of the “best” management for echinococcal cysts is also a clear factor underlying recurrent CE [11]. The purpose of this study was to determine the frequency of CE recurrence, the clinical setting and the risk factors involved in recurrence. Moreover, we studied the treatment applied to these patients and their mortality.

## Methods

The design was a descriptive longitudinal-retrospective study. We reviewed all patients diagnosed with CE according to ICD-9 (code 122–0 to 122–9) criteria admitted at Complejo Asistencial Universitario de Salamanca (CAUSA) between January 1998 and December 2015. CAUSA is a tertiary care hospital, located in western Spain. It covers an area of 12,350 km^2^ with a population of approximately 350,500 individuals [12]. The clinical and epidemiological data were collected after revision of medical records. Diagnosis and classification of CE were assessed according to the criteria proposed by the World Health Organization Informal Working Group on Echinococcosis for CE [6]. Recurrent disease was defined as the appearance of new active cysts after intentional curative therapy, including the reappearance with continuous growth of live cysts at a site of a previously treated cyst or the appearance of new distant disease. To be included in the study, at least one of the following radiological images of the hydatidosis-affected area was to be performed: *i)* abdominal ultrasonography (US) and/or *ii)* computed tomography (CT) in the twelve postoperative months. During follow-up, cysts areas imaged by US and/or CT without a change in size and without evidence of daughter cysts were not considered as recurrence. Patients without follow-up or radiological image, with missing data and who were not recipients of surgery were excluded from the study.

Subsequently, a search was conducted in PubMed from 1966 to January 2016 with *relapses*, *recurrences*, *hydatidosis* and *Echinococcus granulosus* terminus. Clinical cases and non-relevant works were discarded.

### The statistical analysis

The results were expressed as percentages for categorical variables and as the mean and standard deviation (SD) for continuous variables. A chi-square test was used to compare the association between categorical variables, such as clinical and demographics variables, and the measured outcome was expressed as the odds ratio (OR) together with the 95% CI for OR. Continuous variables were compared with Student’s *t*-test, ANOVA or the Mann–Whitney *U* test for two groups, depending on their normal or non-normal distribution. Additionally, we applied the corresponding regression models for multivariate analysis. Recurrence rates were analyzed by Kaplan–Meier method for patients undergoing surgery and apparently disease-free at discharge from hospital. We considered a statistically significant difference from chance at a *p*-value <0.05. All data were analyzed with SPSS 23 (*Statistical Package for the Social Sciences*).

## Results

A total of 571 patients were diagnosed with CE according to ICD-9 (code 122–0 to 122–9) criteria in CAUSA between January 1998 and December 2015. Of these, 343 (60.0%) patients were treated with curative intention (surgery & PAIR [Puncture, Aspiration, Injection, and Re-aspiration]). After exclusion criteria, the study sample was 217 patients. The main epidemiological and clinical data of these patients were shown in Table 1. The mean (±SD) of follow-up was 3.42 ± 3.50 years. The median (25th and 75th percentiles) value was 2.17 years (0.96 and 4.46, respectively). Among the 217 patients, a total of 423 cysts were detected and the cysts per patient mean (±SD) was 1.95 ± 2.66.

**Table 1:** Main epidemiological and clinical data in 217 patients included in the study

	Patients n/N (%)
Age, mean ± SD (range),years ≤ 59 years	52.55 ± 18.20 (5–82) 124/217 (57.1)
Male sex	133/217 (61.3)
Patients from rural areas	148/217 (68.2)
Professional activity-agriculture/livestock	31/217 (14.3)
Contact with animals	60/217 (27.6)
Immigrants	6/217 (2.8)
Comorbidity	73/217 (33.6)
Asyntomatic/casual finding	129/217 (59.4)
Clinical manifestations Mechanics Infectious Allergic	88/217 (40.6) 54/88 (61.3) 35/88 (39.7) 14/88 (15.9)
Eosinophilia (>0.450 × 10^9^ eosinophils/L)	65/217 (29.9)
ELISA (>1/80)	105/217 (48.4)
Cyst location *(multiple response variable)*	
Liver Lung Others/disseminated	193/217 (88.9) 12/217 (5.5) 31/217 (14.2)
No. of cysts, mean ± SD (range) 1 ≥ 2	1.95 ± 2.66 (1–20) 137/217 (63.1) 80/217 (36.9)
Size of the largest cyst, mean ± SD (range),cm ≥ 7 cm ≤ 6.9 cm	8.18 ± 4.35 (1–23) 122/217 (56.2) 95/217 (43.8)
WHO classification CE1 CE2 CE3 CE4 CE5	193/217 (88.9) 10/193 (5.2) 59/193 (30.6) 32/193 (16.6) 30/193 (15.5) 62/193 (32.1)
Treatment	
Combined treatment (Antihelminths & surgical) Surgical technics Surgical treatment complications	141/217 (65.0) 76/217 (35.0) 38/217 (17.5)
Cohort mortality from all causes	13/217 (6.0)^a^
Mortality attributable to hydatidosis	2/217 (0.9)^b^

^a^Exitus ethiology: cardiovascular 1. Cancer 1. Infectious 5. No data 6

^b^Exitus ethiology for *E. granulosus*: Infectious (sepsis) 1. Hemoptysis 1

**Fig. 1:**
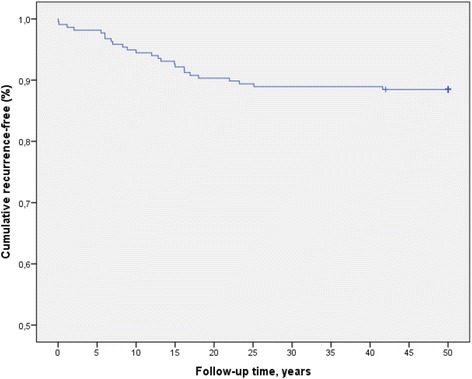
Kaplan-Meier Curve of recurrence-free time in the cohort

**Fig. 2:**
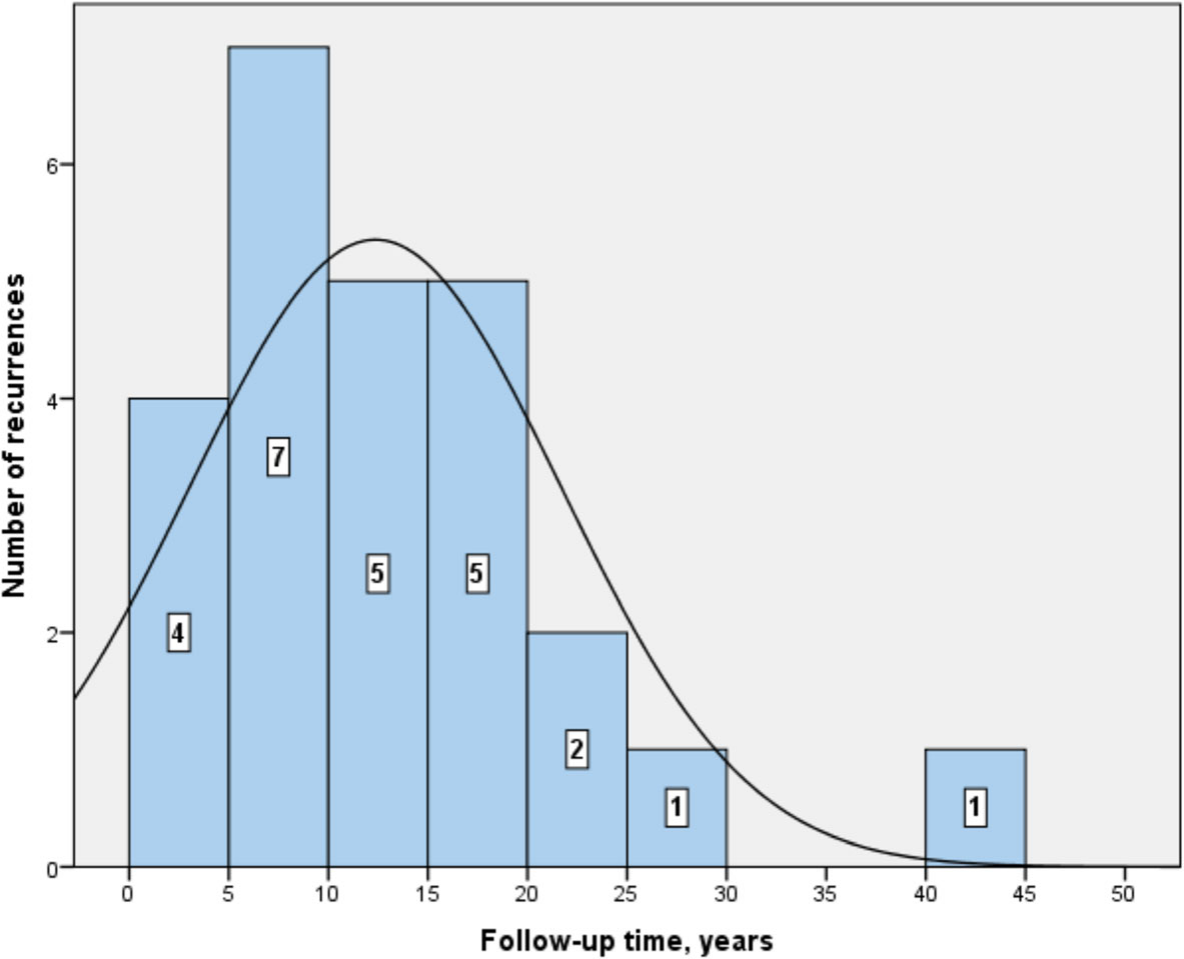
Number of recurrences during follow-up time

A total of 25 (11.5%) patients had CE recurrences during their follow-up. The mean time until diagnosis of CE recurrence was 12.35 years ±9.31 (interval range: 0.02–41.61 years). Four cases were detected in the five first years after surgical intervention, 7 cases between 5 and 10 years of surgical intervention, and 14 cases after 10 years of surgical intervention (Fig. 1). The time trend of recurrences are shown in Fig. 2. In 23 (92%) cases, recurrence appeared next to the surgical site, and only two (8%) recurrences appeared remote to the surgical site (one patient had recurrent CE in the psoas muscle and other in lumbar paravertebral musculature).

Among patients with recurrent CE, 16 (64.0%) were asymptomatic at the moment of diagnosis, with it usually an incidental diagnosis. Nine (36.0%) recurrent cases had clinical manifestations of complicated CE: 6 cases had mechanical complications; two cases had structural displacements; one case had vomica (caused by bronchial fistula); one case had thoracic pain (associated to bronchial fistula); one case was jaundice (by biliary fistula) and one case presented with sciatica (caused by spinal CE). Two patients with recurrent CE presented secondary super-infections pyogenic with suppurative cholangitis and two cases presented urticarial reactions. No patient with recurrent CE died as a consequence of recurrent complicated cyst. The variables associated with recurrence are shown in Table 2. We did not detect any epidemiological variables associated with the occurrence of relapses in patients (*p* > 0.05).

**Table 2:** Study of factors associated with recurrence. Bi-variable analyses between host factors, primary cyst and treatment used with recurrence

Patients with recurrences	n/N (%)	OR (IC95%)	*p*-value*
Host factors			
Age ≤ 59 years	17/124 (13.7)	1.6 (0.6–4.0)	0.244
Male sex	18/133 (13.5)	1.7 (0.6–4.3)	0.243
Patients from rural areas	17/148 (11.5)	0.9 (0.4–2.4)	0.982
Professional activity-agriculture/livestock	2/31 (6.5)	0.4 (0.1–2.1)	0.340
Contact with animals	4/60 (6.7)	0.4 (0.1–1.4)	0.166
Immigrants	0/6 (0.0)	–	–
Comorbidity	7/73 (9.6)	0.7 (0.2–1.8-)	0.526
Cyst primary factors			
Complicated	9/88 (10.2)	0.8 (0.3–1.9)	0.622
Cyst size (≥7 cm)	16/122 (13.1)	1.4 (0.6–3.4)	0.405
Cyst location			
Liver Lung Others/disseminated	19/193 (9.8) 0/12 (0.0) 7/31 (22.6)	0.3 (0.1–0.9) – 2.7 (1.1–7.1)	0.028* – 0.037*
No. of cysts (≥2)	11/80 (13.8)	1.4 (0.6–3.2)	0.432
WHO classification CE1 CE2 CE3 CE4 CE5	19/193 (9.8) 0/10 (0.0) 8/59 (13.6) 4/32 (12.5) 2/30 (6.7) 5/62 (8.1)		0.587
Factors associated to treatment			
Combinated treatment (Antihelminths & surgical)	20/141 (14.2)	2.3 (1.4–6.5)	0.094
Surgical technics	5/76 (6.6)		
Surgical treatment complications	7/38 (18.4)	2.0 (0.7–5.2)	0.142

*p-value of the test: Total patients with treatment with curative intention-follow-up & Recurrences. Statistical significance level of 5% (*p* < 0.05)

In regard to the location of primary CE, recurrence was higher in primary CE located in organs other than the liver or lung [22.6% vs 9.8% vs 0% (OR 2.7 (1.1–7.1) vs 0.3 (0.1–0.9), *p* < 0.05]. Recurrence was higher in patients surgical treated with two or more vs one cyst [13.8% vs 10.2%, OR 1.4 (0.6–3.2)], although these results were not significant (*p* > 0.05) Recurrences were more frequent in primary cysts larger than 7 cm that in smaller cysts, but these differences were not significant [13.1% vs 9.4%, OR 1.4 (95% CI 0.6–3.4), *p* > 0.05]. With respect to WHO classification, relapses were more frequently detected between CE2-CE3 than CE4-CE5 [12/91 (13.2%) vs (7,6%); OR 1.6 (95% CI 0.6–4.3) *p* > 0.05], but these differences were not significant.

We detected the likelihood of recurrence was twice as likely in those patients treated with a combination of anti-helminthic treatments and surgical intervention than patients treated with surgical intervention alone [14.2% vs 6.6%, OR 2.34 (CI 95 0.84–6.52) *p* > 0.05)]. The logistic regression model shows the following clinical pattern: patients with hepatic cysts are treated with surgery alone [Exp (B) = 3.50; 95% CI, 1.20–10.18; *p* < 0.05], whereas patients with localized cysts in organs other than liver or lung receiving combination therapy [Exp (B) = 2.72; 95% CI, 1.03–7.19; *p* < 0.05].

Finally, we evaluated the treatment given to patients with recurrences (Tables 1 and 2). All patients (25, 100%) with recurrences, were treated: 5 (10%) patients with surgical treatment alone, and 20 (80%) patients with a combination of surgical and anti-helminthic treatments (50% with albendazole and 50% albendazole and praziquantel). The mortality in both groups was null.

## Discussion

Cyst echinococcosis (CE) is a parasitic disease highly complex, due mainly to involvement of different organs and tissues and its very slow course (usually over decades). During this large time period, CE may pass from stages active to inactive, and its clinical setting may range from an asymptomatic form to several complications, including fatal disease [4].

The lack of large, longitudinal and controlled studies is due to different factors. First, the chronicity of the disease requires a follow-up of several years to evaluate relapse rates. Second, because there are not tools sensitive enough to allow us to arrive at early diagnosis, and consequently, optimal monitoring after treatment. Finally, its status as a neglected disease contributes to scarce funding for investigations of CE [6]. As a consequence, clinical management has evolved over decades based only on poor-to-moderate quality of evidence and recommendation strengths [6, 7]. Accordingly, major constraints of our work are conditioned by retrospective design. Therefore, the “best” management for CE is still a subject of debate [7]. Besides, in the WHO-IWGE Expert Consensus (World Health Organization-Informal Working Group on Echinococcosis) there is no clear definition of relapse, recurrence and reinfection, which reflects difficulties found in clinical practice.

However, factors such as the introduction of the WHO-IWGE classification for CE or the recent European project HERACLES, have allowed the establishment of a framework that may contribute to advances in the treatment of CE [13].

Today, standard of treatment in CE is based in the use of different surgical techniques with or without chemotherapy and recurrence remains one of the major problems in the management of CE [14] as it can occur with any of the therapeutic methods employed [14]. Although, there are many series of patients with CE, only a few studies specifically analyzed the presence of recurrences and assessed its frequency and surrounding circumstances (Table 3) [8, 14–21].

**Table 3:** Comparative results of different study of recurrence CE

Reference	Type study/period	Country	Number of patients of CE n	Localitation	Number of patients with recurrence CE (%)	Treatment with antihelminths first episode	Morbidity (%)	Mortality (%)	Median Time of follow-up (months)	Interval for recurrences (months)	Risk Factor for recurrences
*Saidi* 1978	Retrospective 1963–1973	Iran	106	159 liver 118 lung 67 others	11.3	ND	ND	8.3	6–36	21.5 ± 14.8	Local spillage
*Kapan* 2006	Retrospective 1998–2003	Turkey	172	172 liver	4.65	Albendazol pre and postoperatively	5.8	0.58	60.5	23.4 ± 5.3	Multiple cysts
*Little* 1988	Retrospective 1980–1986	Australia	39	39 liver	22	No	7.6	0	0–60	30	Rupture hydatid cyst
*Gollackner* 2000	Retrospective 1949–1995	Austria	74	69 liver 3 spleen 2 others	15	50% patients albendazol/mebendazol pre and postoperative	25.0	2.7	93.6 (24–564)	3–240	ND
*El Malki* 2010	Retrospective 1990–2004	Morocco	672	672 liver	8.5	No	ND	ND	24 (10–48)	75 (40–119)	Liver hepatic cyst >3 cyst
*Prousalidis* 2012	Retrospective 1970–2003	Greece	584	436 liver 101 lung 21 peritoneum 12 spleen 13 others	8.7	Albendazol preoperatively and postoperatively	27	ND	58 (48–204)	6–204	Spillage of hydatid cyst missing the cysts pre-intraoperatively incomplete pericystectomy
*Bedioui* 2012	Retrospective 1996–2006	Tunis	391	391 liver	12	ND	ND	ND	51.6	50	Rural origin cyst > 7 cm unilocular cysts
*Akyildiz* 2009	Retrospective 1988–2006	Turkey	412	412 liver	9.2	ND	ND	ND	69.6 (12–180)	24–120	ND
*Atmatzidis* 2005	Retrospective 1982–2001	Greece	109	97 liver 12 others	36	ND	22	2.7	144	84	Chirurgical technique
*Meimarakis* 2009	Retrospective 1982–2004	Germany	10	10 spleen	0	Albendazol/mebendazol	40	0	105.6	0	ND
*Chautems* 2005	Retrospective 1980–1999	Swiss	84	84 liver	0	15% Albendazole postoperative	37	0	103.2	0	ND

*ND* no data

The aim of this work was to determine, in our center, the frequency of recurrence after CE surgery, and the main factors associated with this recurrence. Our work analyzed recurrence using data from clinical records. Therefore, we detected a rate of recurrence above 11%. The rate of recurrence showed by other groups ranged from 0% to 22% in post-surgery [8, 9, 11, 19, 21] and 0%–1.27% in other percutaneous treatments [22]. This wide range reflects several methods employed in these studies, especially with regards to methods used and duration in the follow-up. Despite this, it is probable that all these data on recurrence, including the highest, undervalue the actual incidence.

In regard to the methods used, serological methods did not allow us an optimal follow-up because antibody titers may persist for years after the removal of a cyst. Consequently the relapse must be confirmed by ultrasonography or CT [19]. The differentiation of remaining cavities of effectively treated cysts from locally recurrent disease is difficult, therefore we relied on the accepted imaging marker of the increase in size of the cyst on serial examination, which proved to be effective [19].

Recurrence of CE may be diagnosed after 3 months to 20 years post-surgery, with the mean time ranging 2 and 10 years [9, 10, 17, 18]. In our work, the mean time to detection was lengthy. This is possible because a follow-up prospective after surgery of patients with CE has not been well standardized in our hospital. Therefore, most patients were only prospectively followed-up for one or two years.

In regard to our clinical setting, more than half of our patients were asymptomatic at the moment of diagnosis of relapse. Nevertheless, we also detect patients with CE complications, including pyogenic super-infection, biliary and pleural fistula, or immunological reactions such as a type of urticarial rash. However, there is to highlight that no patient with recurrent CE died as result of these complications. These results are similar to those reported by other authors (Table 3). Consequently, it has been suggested that the postoperative follow-up period should not be shorter than 3 years and should be continued for as long as possible, due the frequently asymptomatic recurrence and the onset of symptoms 3–4 years after surgery [7, 19]. Our results support this strategy, although the efficiency of a screening program in relation to risk and cost has not been established.

Recurrence usually occurs in the same area of the primary CE. Accordingly, in this paper, we detected a local recurrence in more than 90% of cases, and in only two cases was recurrence detected to distance. This fact could be due to the dissemination of the protoscolex from viable CE during surgical procedure by contamination of the surgical bed. However, it is also possible that the dissemination occurs before surgery, especially in complicated CE.

The other objective of our work was to find risk factors associated with recurrence, to implement measure to decrease its risk. In the literature, numerous risk factors have been described, although with important differences between studies [19]. Regarding the factors associated with the host, in our work we found that the epidemiological variables were not associated with recurrences including professional activity–agriculture/livestock or contact with animals. This supports the fact that recurrences are more frequently caused by dissemination before or during surgical procedure than subsequent reinfections.

With respect to primary CE, some characteristics were associated with recurrence. In our study, the location of CE outside of the liver and lung, followed by location in the liver were associated with higher recurrence. Nevertheless, in other series location in the liver [14, 15], in the difficult surgical access [16] or multiple abdominal cysts [14, 15], were variables associated to relapse. Other factors affecting the cyst such as its size, especially in cases larger than 7–10 cm [14, 11] or stages I-II WHO [11] have been associated with recurrence. In our study, we found more frequent relapses in these groups of patients, although these differences were not statistically significant.

In regard to surgery techniques used, the rate of recurrences was higher with laparoscopic surgery, than with open conservative interventions (8.89% vs 3.15%) [16]. Other variables associated with a higher risk or recurrences included: leaving viable material behind during conservative operative interventions [16], incomplete excision of the endocyst, missing the cysts pre- or intraoperatively and incomplete pericystectomy [19] spillage during surgical removal [15, 17, 18, 23]. However, there are no reports showing the association of recurrence and the choice of incision [16]. Two important factors are the surgeon’s practice and experience [15, 24].

In our series, all patients were subjected to open surgery and we do not detect factors increasing the risk of relapse.

Finally, anthelminthic drugs as albendazole or mebendazole, with or without praziquantel, have been used for decrease the risk of relapse in patients undergoing surgery for CE. The results in animal models and humans have shown a reduced recurrence in patients operated with these drugs prophylactically. According to a recommendation of the WHO Informal Working Group on Echinococcosis and other studies, antiparasitic chemotherapy is considered today an indication to prevent secondary echinococcosis and reduce the risk of recurrence [14]. In our work, we detected a higher rate of relapse in patients with combined treatment than with surgery alone. This was possibly a selection bias due to the higher use of combined treatment in complicated cyst CE or greater surgical difficulty.

With respect to the treatment of recurrence, there is no consensus in the literature. Not every patient with documented recurrent active hydatid disease needs to be treated with surgical treatment due to the fact that hydatid disease progresses slowly. Therefore, local recurrence, small cysts in asymptomatic patients with advanced age and/or serious co-morbid conditions are best followed and treated only when complications develop [17, 25]. Haddad et al. suggested that in patients with recurrences smaller than 5 cm in diameter, antihelminthic therapy for 3–6 months was the ideal treatment, and the cysts with difficult location for surgery should be drained percutaneously. In the other hand, there are also reports suggesting that in patients, for whom albendazole treatment for the primary disease failed, treatment of recurrence would also fail. Treatment options for local recurrence are similar to those for the primary disease. However, radical interventions are also suggested in patients with recurrence who previously underwent surgery conservatively. Nevertheless, these radical operations are technically more difficult, and reoperations have higher morbidity and mortality rates [14, 16]. Therefore, the choice between conservative vs radical interventions depends on a number of factors, such as location, size and stage of the cysts/lesions, and availability of therapeutic options in each health center. In our work, all patients with recurrent CE were subjected to surgical intervention and the mortality was null, including complicated CE cases. This supports the notion that surgical treatment in well selected cases is associated with good outcomes.

## Conclusions

CE recurrence remains one of the major problems in the management of hydatid disease. It can occur decades after surgery, and in the clinical setting is usually diagnosed only incidentally. However, primary cyst can be detected as a complicated cyst. The main risk factor associated with relapse is the location of the primary cyst outside of the liver and lung followed by liver cysts. Mortality in this group of patients was null and surgical intervention of recurrent cyst in well-selected cases is associated with good outcomes.

## Abbreviations

CAUSA: Complejo Asistencial Universitario de Salamanca
CE: Cystic echinococcosis
WHO-IWGE: World Health Organization-Informal Working Group on Echinococcosis
